# Defining regorafenib as a senomorphic drug: therapeutic potential in the age-related lung disease emphysema

**DOI:** 10.1038/s12276-023-00966-6

**Published:** 2023-04-03

**Authors:** Jung-Jin Park, Kwangseok Oh, Gun-Wu Lee, Geul Bang, Jin-Hee Park, Han-Byeol Kim, Jin Young Kim, Eun-Young Shin, Eung-Gook Kim

**Affiliations:** 1 Department of Biochemistry, Chungbuk National University College of Medicine, Cheongju, 28644 Korea; 2 Research Center for Bioconvergence Analysis, Korea Basic Science Institute, Ochang, 28119 Korea

**Keywords:** Senescence, Growth factor signalling, Chronic inflammation

## Abstract

Senescence, a hallmark of aging, is a factor in age-related diseases (ARDs). Therefore, targeting senescence is widely regarded as a practicable method for modulating the effects of aging and ARDs. Here, we report the identification of regorafenib, an inhibitor of multiple receptor tyrosine kinases, as a senescence-attenuating drug. We identified regorafenib by screening an FDA-approved drug library. Treatment with regorafenib at a sublethal dose resulted in effective attenuation of the phenotypes of βPIX knockdown- and doxorubicin-induced senescence and replicative senescence in IMR-90 cells; cell cycle arrest, and increased SA-β-Gal staining and senescence-associated secretory phenotypes, particularly increasing the secretion of interleukin 6 (IL-6) and IL-8. Consistent with this result, slower progression of βPIX depletion-induced senescence was observed in the lungs of mice after treatment with regorafenib. Mechanistically, the results of proteomics analysis in diverse types of senescence indicated that growth differentiation factor 15 and plasminogen activator inhibitor-1 are shared targets of regorafenib. Analysis of arrays for phospho-receptors and kinases identified several receptor tyrosine kinases, including platelet-derived growth factor receptor α and discoidin domain receptor 2, as additional targets of regorafenib and revealed AKT/mTOR, ERK/RSK, and JAK/STAT3 signaling as the major effector pathways. Finally, treatment with regorafenib resulted in attenuation of senescence and amelioration of porcine pancreatic elastase-induced emphysema in mice. Based on these results, regorafenib can be defined as a novel senomorphic drug, suggesting its therapeutic potential in pulmonary emphysema.

## Lung disease: existing drug could block emphysema progression

An existing drug that can inhibit the deterioration of cell activity due to aging could provide a novel treatment for the debilitating lung disease emphysema. Aging-related diseases, including emphysema, are exacerbated by cellular senescence, the loss of cell division and growth due to aging. ‘Senomorphic’ drugs could halt senescence and stop the progression of aging-related diseases. Eung-Gook Kim and Eun-Young Shin at Chungbuk National University, Cheongju, South Korea, and co-workers screened an approved drug library for senomorphic drug candidates to treat emphysema. Their search highlighted regorafenib, a drug that is used to treat colorectal and gastrointestinal cancers. In experiments on mouse models, regorafenib exhibited a strong inhibitory effect on different types of senescence by regulating the expression of multiple proteins and inhibiting key signaling pathways. The drug blocked emphysema progression in the lungs of mice.

## Introduction

Cellular senescence is characterized by irreversible growth arrest, increased senescence-associated β-galactosidase (SA-β-Gal) activity, the senescence-associated secretory phenotype (SASP), and typical hyperadhesive morphological changes^1,2^. Senescence occurs as a cellular response to diverse types of stresses. Senescence, a hallmark of aging, is a factor in aging and age-related diseases (ARDs)^3,4^, particularly lung diseases, including chronic obstructive pulmonary disease (COPD), whose major types are emphysema and chronic bronchitis^5,6^. The SASP, which is regarded as a major pathogenic mechanism, exerts an effect on surrounding cells through the secretion of many inflammatory mediators and growth factors^7^. Targeting senescence has been found to result in attenuated progression of ARDs; thus, it is widely accepted as a practicable method for use in the treatment of ARDs^8,9^. Accordingly, active clinical investigation of senolytics capable of killing senescent cells is currently underway^10^.

Although replicative senescence induction was originally recognized as a method for establishing a cellular model of senescence^1^, a relatively long culture time is required before experiments can be conducted. Despite this shortcoming, this model has long been utilized in the effort to understand the molecular mechanisms of and screen chemicals that can mediate the suppression of senescence. Subsequent studies resulted in the discovery of stimulus-induced premature senescence (SIPS), which can be induced to establish a more convenient model of senescence, where senescence occurs within several days to 2 weeks^11^. Typical stimuli used for the induction of SIPS include oncogene expression and treatment with chemotherapeutic drugs such as doxorubicin^12^. Downregulation of beta p21-activated kinase (PAK) interacting exchange factor (βPIX), a focal adhesion protein, leads to induction of senescence in vitro and in vivo via activation of integrin signaling^13^. The expression of βPIX decreases with age, suggesting a causal effect on aging-related senescence. Thus, βPIX-depleted cells can be employed as a new cellular model for screening antisenescence modulators. This model may also have an additional advantage. Integrin is a therapeutic target in aging/senescence-associated diseases such as lung fibrosis^14^ and emphysema^15^ because aberrant integrin signaling is closely related to their development. Considering that the level of βPIX decreases with age in the lungs of mice and that it plays a causative role in senescence, the βPIX depletion model may serve as a valuable resource to delineate the pathogenic mechanism of integrin signaling.

Convergence of senescence signaling occurs in the p53/p21^WAF1^ and p16^INK4a^/pRb pathways;^16^ however, the mediation of diverse senescence phenotypes also involves other signaling pathways, such as the receptor tyrosine kinase, integrin adhesion receptor, and transforming growth factor (TGF)-β signaling pathways. Distinct cell- and tissue-specific phenotypes are generated through spatiotemporal regulation of these signaling pathways and crosstalk among them^17^. A serine/threonine protein kinase (AKT)/mammalian target of rapamycin (mTOR) signaling downstream of these growth factor receptors plays a critical role in the regulation of many biological processes, including aging and senescence^18,19^. mTOR, a serine/threonine kinase, is an important component of two distinct complexes, mTORC1 and mTOR complex 2 (mTORC2)^18^. Findings from earlier studies demonstrated that mTOR is a critical target of rapamycin, and inhibition of rapamycin was reported to result in an extended lifespan in model organisms^20,21^. Findings from one study demonstrated the sensitivity of mTORC1 but not mTORC2 to short-term treatment with rapamycin^22^. Thus, extensive study of the role of mTORC1 in regard to aging and senescence, in addition to its roles in cell growth, metabolism, and autophagy, has been conducted^23,24^. Downstream kinases such as AKT, protein kinase C (PKC), and serum/glucocorticoid regulated kinase 1 (SGK1) are involved in the regulation of cell survival, cytoskeletal organization, and sodium transport by mTORC2^25^. However, the understanding of the role of mTORC2 in cellular senescence is still limited.

Considering that the development of a new drug involves massive effort, repurposing FDA-approved drugs appears to be a sensible strategy. In particular, application of this approach to diseases with unmet clinical needs seems to be a worthwhile endeavor. Inhibitors that target kinases, particularly multiple receptor tyrosine kinases (RTKs), are currently utilized as therapeutic drugs^26^. Most of these inhibitors are utilized as anticancer drugs and exhibit antitumorigenic activities through induction of cell death and/or inhibition of cell invasion and metastasis. Of particular importance, they exhibit distinct biological activities at low concentrations that do not induce significant cell death, and can therefore be employed for other uses such as targeting senescence and treating ARDs. One example is nintedanib, which is prescribed for the treatment of idiopathic pulmonary fibrosis, a fatal age-related lung disease^27^. Regorafenib, which is also an inhibitor of multiple RTKs, is approved for the treatment of patients with metastatic colorectal and hepatocellular carcinoma and gastrointestinal stromal tumors^28,29^. The beneficial effects of regorafenib have been reported in recent studies in animal models of Alzheimer’s disease, bleomycin-induced fibrosis, and pulmonary hypertension, suggesting its broad application potential^30–32^.

Our purpose in the current study was to identify one or more novel modulators of senescence using a βPIX depletion-induced model of senescence as a system for screening. Regorafenib was identified as a senescence-attenuating drug. A porcine pancreatic elastase (PPE)-induced model of emphysema in mice was used to further evaluate the therapeutic potential of regorafenib in the treatment of pulmonary emphysema, a senescence and age-related lung disease. To gain mechanistic insights, a rigorous analysis including a proteomics study was performed to identify the senescence-related target proteins of regorafenib and to examine the downstream pathways that might provide an explanation for its in vitro and in vivo effects.

## Materials and methods

### Materials

The FDA-approved drug library (L1300) and regorafenib were purchased from Selleckchem (Houston, TX). Porcine pancreatic elastase (PPE) was obtained from Elastin Products Company, Inc. (Owensville, MO). Invivofectamine 3.0, Lipofectamine 3000, RNAiMAX, fetal bovine serum (FBS), and Opti-Minimum Essential Medium (MEM) were purchased from Thermo Fisher Scientific, Inc. (Waltham, MA). PCR primers and siRNAs were obtained from Bioneer (Daejeon, Korea) and Thermo Fisher Scientific, Inc., and a list of their sequences is provided in the supplementary materials. A summary of the primary and secondary antibodies used is provided in the supplementary materials. Male C57BL/6 mice were obtained from Daehan Bio Link (DBL) (Chungbuk, Korea). Animals received sufficient food and water. All experimental protocols were approved by the Chungbuk National University Animal Care and Use Committee^33^. Detailed information regarding the materials used in this study is provided in the supplementary materials.

### Cell culture and cellular senescence models

IMR-90 cells were cultured in MEM supplemented with 10% fetal bovine serum (FBS) and antibiotics in a humidified 5% CO_2_ incubator at 37 °C. To establish βPIX depletion-induced senescence, IMR-90 cells were incubated with the siPIX RNA–Lipofectamine RNAiMAX complexes according to the manufacturer’s instructions^13^. An SA-β-Gal staining assay or Western blotting was performed after two or 3 days. Following treatment with 400 nM doxorubicin for 2 h, IMR-90 cells were washed and incubated with fresh culture medium for 3 days to establish doxorubicin-induced senescence. Young IMR-90 cells at passage numbers 10~12 were cultured to passages 45~46 to induce replicative senescence.

### SA-β-Gal staining

Fixation of IMR-90 cells was performed by application of 2% formaldehyde and 0.2% glutaraldehyde for 10 min prior to incubation in SA-β-Gal staining solution (1 mg/ml 5-bromo-4-chloro-3-indolyl-beta-d-galactopyranoside (X-gal), citric acid/sodium phosphate buffer (pH 6.0), 5 mM potassium ferricyanide, 5 mM potassium ferrocyanide, 150 mM NaCl, and 2 mM MgCl_2_) at 37 °C for 20-24 h. For cell counting, cells were incubated in 0.5 μg/ml DAPI solution for 10 min and were then washed with PBS. Cellular senescence was scored as the percentage of SA-β-Gal-positive cells (stained blue) relative to the total cell number.

For tissue staining, animals were anesthetized, and perfusion was performed using phosphate-buffered saline (PBS). After embedding in OCT compound, lung tissues were immediately sliced into 10 μm sections using a cryotome (Leica Biosystems, Germany). Following fixation with 0.5% glutaraldehyde and washing with PBS, the tissue sections were incubated in SA-β-Gal staining solution (pH 5.8) at 37 °C for 20-24 h. Then, following incubation with safranin O solution, the tissue slides were mounted with Vectashield mounting medium (Vector Laboratories, Inc., Burlingame, CA).

### Cell proliferation assay

The cell proliferation assay was performed as previously described^13^. After plating in 6-well plates and transfection with the siPIX or siCtrl RNAs, IMR-90 cells were treated with or without regorafenib. Harvesting of cells using trypsin and staining with crystal violet solution was performed at each time point. A hemocytometer was used to count the cells.

### In vivo transfection of siRNAs

Preparation of the Invivofectamine–siRNA (1 mg/kg) complexes was performed according to the manufacturer’s instructions. The intratracheal delivery method was used for the introduction of siRNAs into the lungs of male C57BL/6 mice (12 weeks old)^13^. Briefly, following anesthetization of mice, a catheter (1 inch, 22 gauge Safelet IV) with a blunted needle was inserted into the trachea under direct visualization. After 3 weeks, regorafenib (5 mg/kg) was administered orally once a day for 2 weeks.

### Quantitative real-time PCR (qRT‒PCR)

Total RNA extraction was performed using a spin-RNA kit, and complementary DNA (cDNA) synthesis was performed using a cDNA synthesis kit (iNtRON, Sungnam, Korea) according to the manufacturer’s protocol. Quantitative RT-PCRs was performed in triplicate using IQ SYBR Green Supermix (Bio-Rad). CFX Manager software (Bio-Rad) was used for setup of the experiment and analysis of the data. All values were normalized to those of actin. A list of primers used in this study is provided in the Supplementary Materials.

### Western blotting

A list of the primary antibodies is provided in the Supplementary Materials. Lysis of cells and extraction of proteins were performed in RIPA buffer [50 mM Tris, pH 8.0; 150 mM NaCl; 1% Nonidet P-40; 0.5% sodium deoxycholate; 0.1% sodium dodecyl sulfate (SDS)] containing phosphatase inhibitor and protease inhibitor cocktails. Equal amounts of protein were separated on an SDS-polyacrylamide gel and transferred to a polyvinylidene fluoride membrane. After blocking the membrane in milk containing Tris-buffered saline and Tween 20 (TBS-T; 50 mM Tris, 150 mM NaCl, and 0.2% Tween 20) for 1 h, the membranes were incubated with specific primary and horseradish peroxidase (HRP)-conjugated secondary antibodies. The enhanced chemiluminescence (ECL) system was used for the detection of immunoreactive bands using ChemiDoc imaging systems (Bio-Rad Laboratories, Inc.).

### Antibody array analysis

To assess the SASP and phosphorylation of receptors and kinases, the following antibody arrays were used: a Human Inflammation Antibody Array (Cat #ab134003, Abcam, MA), Proteome Profiler Human Phospho-Receptor Tyrosine Kinase Kit (Cat #ARY001B, R&D Systems, MN), and Proteome Profiler Human Phospho-Kinase Array Kit (Cat #ARY003C, R&D systems). Experiments were conducted according to the manufacturers’ protocols. Briefly, antibody array membranes were blocked using 10% FBS in TBS-T for 1 h prior to incubation with cell lysates at 4 °C overnight. The membranes were washed with TBS-T prior to incubation with paired biotinylated detector antibodies and streptavidin–HRP. The ECL system was used for the detection of immunoreactive spots using ChemiDoc imaging systems (Bio-Rad Laboratories, Inc. CA). ImageJ software was used for quantification of immunoreactive spot intensities.

### Immunohistochemistry

After fixation with formalin, tissues were embedded in paraffin. Tissue sections with a thickness of 4 μm were deparaffinized, and antigen retrieval was carried out in 10 μg/ml proteinase K solution or 10 mM sodium citrate buffer (pH 6.0). Following antigen retrieval, the slides were incubated in blocking buffer (1% BSA, 0.1% cold fish skin gelatin, 0.5% Triton X-100, 0.05% sodium azide, 0.01 M PBS) for 1 h. The slides were then incubated in the primary antibody solution at 4 °C overnight prior to washing three times with PBS-T (PBS containing 0.1% Tween 20). For diaminobenzidine HCl (DAB) staining, the slides were incubated in methanol with 0.3% hydrogen peroxide at 20 °C for 20 min, incubated with a biotin-conjugated secondary antibody for 1 h at 20 °C, and incubated with peroxidase-conjugated streptavidin for 30 min at 20 °C. Finally, detection of signals was performed using the DAB substrate, and analysis was performed using ImageJ (Color Deconvolution plugin) and GraphPad Prism 8.0 (GraphPad, La Jolla, CA) software.

### TMT 10-plex labeling and LC‒MS/MS analysis

Labeling of trypsin-digested peptides was performed using 10-plex TMT reagent according to the manufacturer’s instructions (Thermo Fisher Scientific Inc., MA). Reg-treated peptides from siCtrl-, siPIX-, and siPIX-transfected cells were labeled with TMT reagents. After labeling, all TMT-labeled peptides were combined and dried using a SpeedVac. Fractionation of TMT-labeled peptides was performed by elution in an increasing acetonitrile step gradient using a high-pH reversed-phase peptide fractionation kit (Thermo Fisher Scientific Inc.). After vacuum drying, nine samples of fractionated peptides were dissolved in 20 μL of water containing 0.1% formic acid for LC‒MS/MS analysis. LC‒MS/MS analysis was performed using an EASY-nLC 1200 UPLC system (Thermo Fisher Scientific Inc.) coupled to an Orbitrap Fusion Lumos Tribrid mass spectrometer (Thermo Fisher Scientific Inc.). Mobile phases A and B were composed of 0 and 80% acetonitrile containing 0.1% formic acid, respectively. An LC gradient of 2 h at a flow rate of 250 nL/min was applied for peptide separation. During separation, data-dependent mode was used for operation of the Orbitrap Fusion Lumos mass spectrometer. Acquisition of MS2 spectra was performed using the Orbitrap mass analyzer at a resolution of 60,000 with high-energy collision dissociation (HCD) at 37.5% normalized collision energy. Identification and quantification of MS/MS spectra was performed using Integrated Proteomics Pipeline software with the UniProt human database (released on 06-02-2020). All raw MS data files generated in this study have been deposited in the MassIVE repository with identifier PXD036234.

### Generation of an emphysema animal model

Mice (8 weeks old) were randomly divided into three groups: saline, porcine pancreatic elastase (PPE, 0.2 U/kg), and PPE plus regorafenib (5 mg/kg). An intraperitoneal (IP) injection of Zoletil plus Rompun (Zoletil:Rompun:saline = 1:1:8) was administered to anesthetize the mice. PPE was administered by intranasal instillation. After 2 weeks, regorafenib was administered to the mice in the PPE plus regorafenib group by oral gavage (5 mg/kg once a day for 1 week). Following collection of the lungs, they were subjected to a lung function test, immunohistochemical staining, and Western blotting.

### Measurement of the mean chord length (Lm)

Lung tissues were prepared as paraffin sections and were then stained with hematoxylin and eosin (H&E). Five random fields per mouse were photographed under a microscope using a digital camera (200x magnification), and analysis was performed using ImageJ software. Estimation of the mean chord length (Lm) in the airspace was based on the average size of alveoli^34^.

### Lung function test

The lung function test was performed using the flexiVent FX system (SCIREQ Inc., Montreal Qc, Canada)^33^. After being anesthetized, mice were connected to the flexiVent system via a catheter inserted in the airway. Measurement of various perturbation parameters was performed using the flexiVent system according to the manufacturer’s instructions. Calculation and quantitative analysis of each parameter was based on an average of three measurements per mouse.

### Statistical analysis

Data are expressed as the means ± standard errors of the mean (SEMs). Representative data from at least three independent experiments were analyzed. *P* < 0.05 was considered to indicate statistical significance. Assessment of statistical significance was performed using unpaired Student’s *t*-test (*t*-test), one-way ANOVA, and the Wilcoxon signed–rank test, and the results are provided in the figure legends and graphs (GraphPad Prism software).

## Results

### Identification of regorafenib as a senescence-attenuating drug

**Fig. 1:**
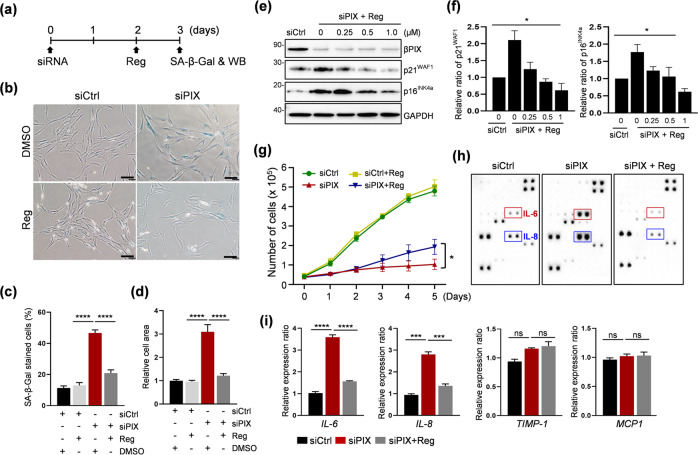
Characterization of regorafenib as an attenuator of senescence in IMR-90 cells. **a** Experimental scheme. Transfection of cells with siPIX RNAs was performed to induce senescence. After 2 days, the cells were treated with regorafenib (Reg, 1 μM) or DMSO for 24 h, followed by analysis using an SA-β-Gal staining assay and Western blotting. **b** Representative images of SA-β-Gal staining. **c** Quantification of SA-β-Gal-stained cells. *N* ≥ 300 cells per group from three independent experiments. **d** Quantification of cell morphological changes. *N* ≥ 40 cells per group from three independent experiments. **e** Representative images of Western blotting using antibodies against p16^INK4a^, p21^WAF1^, βPIX, and GAPDH. **f** Quantification of the data in (**e**). The expression of the p16^INK4a^ and p21^WAF1^ proteins was normalized to that of GAPDH. One-way ANOVA. **g** Cell proliferation assay. Counting of siRNA-transfected cells with or without Reg (1 μM) was performed at the indicated times. *p = 0.0313, Wilcoxon signed–rank test. **h** Representative images of human inflammation antibody array membranes. **i** qRT‒PCR was performed for confirmation (**h**). The mRNA expression of *IL-6*, *IL-8*, *TIMP1*, and *MCP1* was quantified. The error bars indicate the mean ± SEM of three independent experiments. *p < 0.05, ***p < 0.001, ****p < 0.0001, ns, not significant, *t*-test.

Screening of 1018 drugs included in an FDA-approved drug library was performed using a βPIX knockdown model of cellular senescence in IMR-90 cells, human lung fibroblasts, to identify senescence-attenuating drugs^13^. Several drugs were identified through two rounds of screening; of these, screening based on the activity of SA-β-Gal demonstrated that regorafenib was the most efficient inhibitor. Regorafenib, an anticancer drug, is utilized in the treatment of metastatic colorectal and gastrointestinal stromal tumors^35^. Regorafenib is capable of blocking downstream signaling of vascular endothelial growth factor receptor (VEGFR), platelet-derived growth factor receptor (PDGFR), and fibroblast growth factor receptor (FGFR) due to its strong inhibitory activity against multiple receptor tyrosine kinases^36^. As shown in Fig. 1a, cytotoxicity (Supplementary Fig. 1a) and apoptosis (Supplementary Fig. 1b) could not be observed after treatment with concentrations of regorafenib up to 1.0 µM. Therefore, 1.0 µM regorafenib was used as the maximum concentration in the cellular experiments. Lower levels of SA-β-Gal activity were observed in regorafenib-treated cells than in βPIX-depleted cells (Fig. 1b; quantified in c). Assessment based on the cell area showed that treatment with regorafenib also resulted in a reduction in the size of senescent cells (Fig. 1d). A dose-dependent effect of regorafenib on the levels of p21^WAF1^ and p16^INK4a^, which were increased by depletion of βPIX, was observed (Fig. 1e; quantified in f). Thus, treatment with regorafenib resulted in partial restoration of the proliferative ability of senescent cells without an increase in the cell number over the 5-day culture period (Fig. 1g). Next, we examined the effect of regorafenib on the SASP. A cytokine array was used for this purpose (Fig. 1h). Among the numerous components of the SASP, notable production of both interleukin 6 (IL-6) and IL-8 was observed in βPIX-depleted cells and was reversed by treatment with regorafenib (Fig. 1h–i). In contrast, no significant changes were observed with regard to other SASP factors, such as *TIMP-1* and *MCP1*, a monocyte chemotactic factor. Taken together, these findings indicate that treatment with regorafenib results in attenuated senescence of IMR-90 cells induced by depletion of βPIX.

Next, we attempted to determine whether regorafenib has a similar function in other types of senescence, doxorubicin-induced senescence and replicative senescence (Supplementary Fig. 2). As shown in Supplementary Fig. 2a, treatment with doxorubicin, a DNA-damaging agent, resulted in an increase of ~60% in the number of SA-β-Gal-positive cells (Supplementary Fig. 2b; quantified in c). Treatment with regorafenib resulted in a reduction in this increase to 38% positivity. The levels of the upregulated factors p21^WAF1^ and p16^INK4a^ showed a dose-dependent decrease after treatment with regorafenib (Supplementary Fig. 2d; quantified in e). The senescence-attenuating effect of regorafenib was further examined in IMR-90 cells at the 45 population doubling level. Old (high-passage) cells were treated with regorafenib for 1 day (Supplementary Fig. 2f). At this high passage number, ~50% of IMR-90 cells showed positivity for SA-β-Gal (Supplementary Fig. 2g, h). Markedly increased levels of both p21^WAF1^ and p16^INK4a^ were also observed (Supplementary Fig. 2i, j). These features are consistent with those of old senescent cells and were ameliorated by treatment with regorafenib (Supplementary Fig. 2f–j). Taken together, these results support the potential application of regorafenib as a senomorphic drug.

### Regorafenib attenuated βPIX depletion-induced senescence in the lungs of mice

**Fig. 2:**
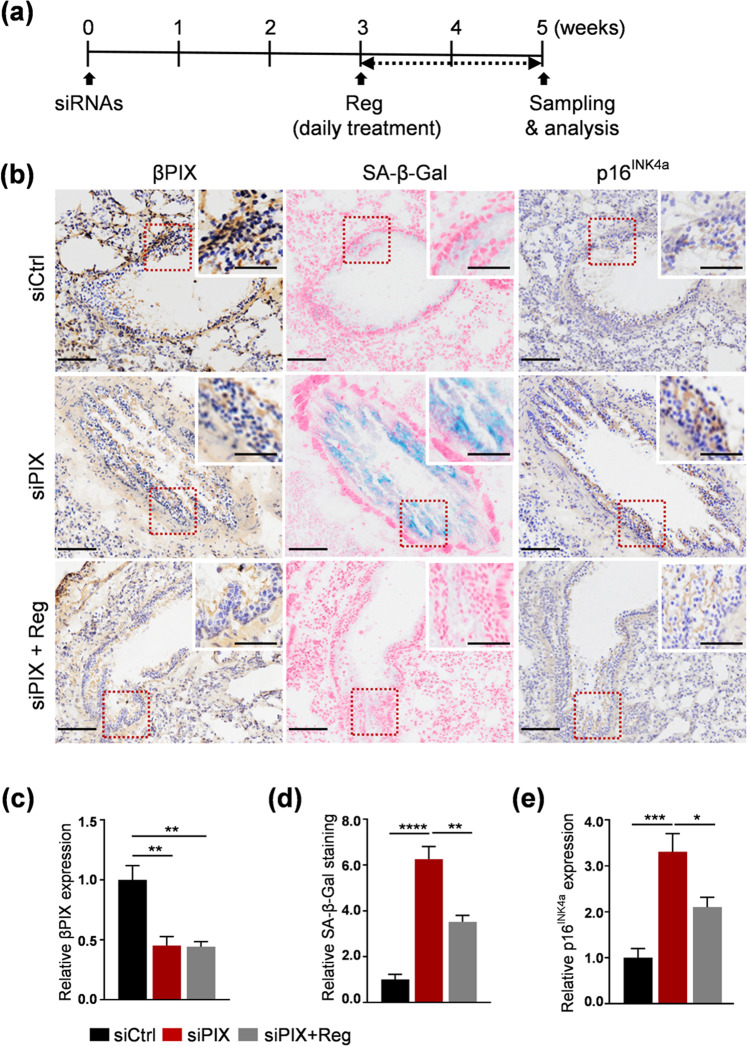
Treatment with regorafenib resulted in attenuation of βPIX depletion-induced senescence in the lungs of mice. **a** Experimental scheme. SiRNAs were administered to mice by intratracheal injection. After 3 weeks, Reg (5 mg/kg) was administered orally once a day for 2 weeks. SA-β-Gal staining and immunohistochemistry (IHC) were performed on lung tissues. **b** Representative images of bronchioles and surrounding tissues. **c** ~ **e** Quantification of the data in **b**. Quantification of βPIX expression (**c**), SA-β-Gal staining (**d**), and p16^INK4a^ expression (**e**). *N* = 5 mice per group. The error bars indicate the means ± SEMs, *p < 0.05, **p < 0.01, ***p < 0.001, ****p < 0.0001, *t*-test. Scale bars, 50 μm.

Findings from previous studies demonstrated that senescence can be induced in the lungs of young mice by depletion of βPIX^13^. This model of senescence was established by intratracheal delivery of liposomal βPIX siRNA (siPIX) to examine the in vivo effects of regorafenib. To exclude the possibility that regorafenib may function through a senolytic effect in mice, we determined whether regorafenib induces apoptotic cell death. For this purpose, regorafenib was orally administered at dosages of up to 15 mg/kg/day for 2 weeks (Supplementary Fig. 3a). The basal levels of cleaved PARP and cleaved caspase 3 were detected in the lung even after administration of 15 mg/kg/day (Supplementary Fig. 3b). Consistent with this result, the TUNEL assay showed no significant differences between the control and regorafenib-treated groups (Supplementary Fig. 3c, d). Considering these results collectively, we concluded that regorafenib is not senolytic in the lungs at the dosage of 5 mg/kg/day used in the present study. As shown in the schematic (Fig. 2a), 3 weeks after instillation of siPIX, regorafenib (5 mg/kg/day) was administered orally for 2 weeks. Because siPIX did not show an equal distribution in the lungs, our examination of the senescence effect was focused on the area where decreased levels of βPIX were detected by immunohistochemistry. Consistent elevation of senescence markers, namely, SA-β-Gal staining and the level of p16^INK4a^, was observed in βPIX-depleted bronchioles compared to control bronchioles (Fig. 2b; quantified in c–e). Treatment with regorafenib resulted in alleviation of these effects that were induced by depletion of βPIX, as determined by the diminished SA-β-Gal staining (Fig. 2d) and p16^INK4a^ level (Fig. 2e). These results indicate a similar in vivo function of regorafenib as an attenuator of senescence.

### Regorafenib regulates the expression of multiple senescence-related proteins

**Fig. 3:**
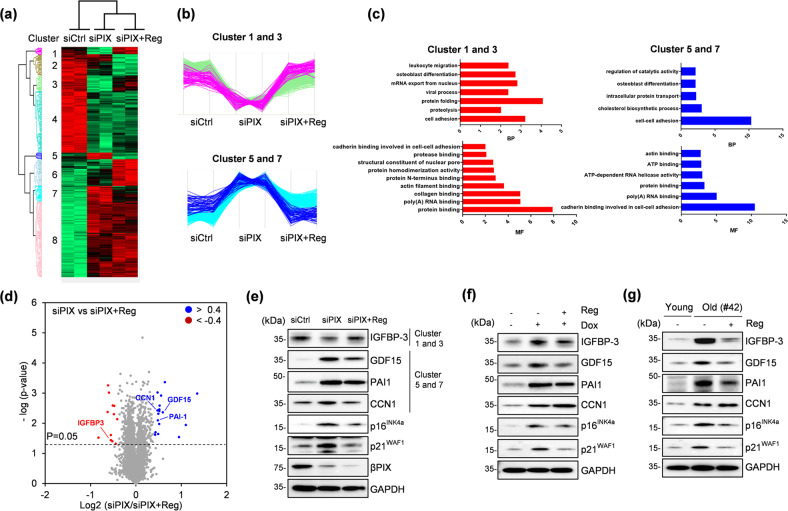
Proteomic analysis of differentially expressed proteins after treatment of βPIX-depleted senescent IMR-90 cells with regorafenib. **a** Heatmaps of the identified proteins (*N* = 2,255) in siCtrl, siPIX and siPIX plus Reg (1 μM) cells. *p* < 0.05, ANOVA for multisample tests. **b** Grouping of protein clusters based on their expression patterns in response to treatment with siRNA and Reg. **c** Pathway analysis of proteins grouped into Clusters 1 and 3 and Clusters 5 and 7. **d** Volcano plot of the differentially expressed proteins. A log2-fold change of >0.4 or < –0.4 (*p* < 0.05) was regarded as significant. Blue, upregulated proteins; red, downregulated proteins; gray, non-differentially expressed proteins. **e** Western blot analysis of differentially expressed proteins. Immunoblotting of lysates with the indicated antibodies specific for the hit proteins was performed. **f**, **g** Western blot analysis of the hit proteins that responded to Reg (1 μM) in doxorubicin-induced (**f**) and replicative (**g**) senescent cells.

Tandem mass tag (TMT)-labeling proteomic analysis was performed in IMR-90 cells to gain mechanistic insights into the senescence-attenuating effect of regorafenib (Fig. 3 and Supplementary Fig. 4). A total of 4990 proteins were identified from the TMT experiment (Supplementary Table 1). The proteins (*N* = 2,225) that showed altered expression (*p*-value < 0.05) in response to siPIX and regorafenib are shown in a heatmap (Fig. 3a). Two distinct groups of clusters were identified from this map; the group containing Clusters 1 and 3 and the other group containing Clusters 5 and 7 showed opposite responses to siPIX and regorafenib, respectively (Fig. 3b). We focused on the proteins from these clusters as potential targets of regorafenib that might provide an explanation for its senescence-attenuating effect. These proteins were grouped according to their biological functions to obtain a better understanding (Fig. 3c). In Clusters 1 and 3, proteins involved in protein folding and binding were ranked highest, whereas in Clusters 5 and 7, markedly affected expression of cell‒cell adhesion and cadherin binding-related proteins was observed, indicating a biological function of the adhesion protein βPIX. The regorafenib-responsive proteins are visualized in a volcano plot (Fig. 3d and Supplementary Table 2). Among these, proteins with a log2-fold change in expression of >0.4 or < −0.4 (*p*-value < 0.05) were regarded as significantly altered proteins and denoted by red and blue dots, respectively (Fig. 3d). A list of 13 upregulated proteins and 18 downregulated proteins is shown in Table 1. Next, the expression of senescence-related proteins among regorafenib-responsive proteins, namely, insulin-like growth factor binding protein-3 (IGFBP-3)^37^, growth differentiation factor 15 (GDF15)^38^, plasminogen activator inhibitor-1 (PAI-1)^39^, and cellular communication network factor 1 (CCN1)^40^, was confirmed. Samples that were prepared independently from cells with siPIX-induced senescence, cells with doxorubicin-induced senescence and old senescent cells were used in this experiment (Fig. 3e–g). Immunoblotting was performed on cells in a similar senescent state where significant upregulation of p16^INK4a^ and p21^WAF1^ was observed. Treatment with regorafenib resulted in enhanced expression of IGFBP3 in siPIX-treated cells (Fig. 3e); however, its expression was reduced in doxorubicin-treated and old senescent cells (Fig. 3f, g). Decreased levels of GDF15 and PAI-1 were observed under the three different senescence conditions. Downregulation of CCN1 was observed in siPIX-treated cells (Fig. 3e); however, doxorubicin-treated and old senescent cells showed no significant changes in CCN1 expression (Fig. 3f, g). Taken together, these results from examination of cells under three senescence conditions indicate that GDF15 and PAI-1 are shared senescence-related targets of regorafenib.

**Table 1:** List of proteins identified in the proteomic analysis: siPIX vs. siPIX+Reg.

UniProt ID	Gene name	Fold change Log2(siPIX+Reg/siPIX)	Log (*p* value)
**DOWN**			
P13726	F3	−1.35	2.99
P35527	KRT9	−1.10	1.95
P04264	KRT1	−0.95	1.55
Q01581	HMGCS	−0.64	3.37
Q99988	GDF15	−0.59	2.36
O94851	MICAL2	−0.55	2.92
Q4L180	COL4A3BPIP	−0.52	2.59
P62987	UBA52	−0.52	2.46
P02795	MT2A	−0.51	2.41
Q96NE9	FRMD6	−0.51	1.98
Q96PD2	DCBLD2	−0.49	2.32
Q8IVM0	CCDC50	−0.49	1.65
P05121	PAI-1	−0.48	2.10
P49137	MAPKAPK2	−0.48	3.02
O00622	CCN1	−0.48	2.43
O14867	BACH1	−0.44	1.18
P48060	GLIPR1	−0.43	1.71
Q15014	MORF4L2	−0.42	1.62
**UP**			
Q96C12	ARMC5	0.82	1.53
O60462	NRP2	0.62	2.38
Q53EL6	PDCD4	0.61	3.26
Q8WUJ3	CEMIP	0.59	3.02
P17936	IGFBP-3	0.55	1.44
P00736	C1R	0.55	1.61
Q9NVM1	EVA1B	0.52	1.41
Q8IZV5	RDH10	0.51	2.59
P54764	EPHA4	0.48	2.30
Q86YT6	MIB1	0.48	2.58
P16403	H1-2	0.47	1.22
Q96PE1	ADGRA2	0.45	1.32
Q9P246	STIM2	0.41	2.14

### Regorafenib inhibited multiple senescence-related signaling pathways

**Fig. 4:**
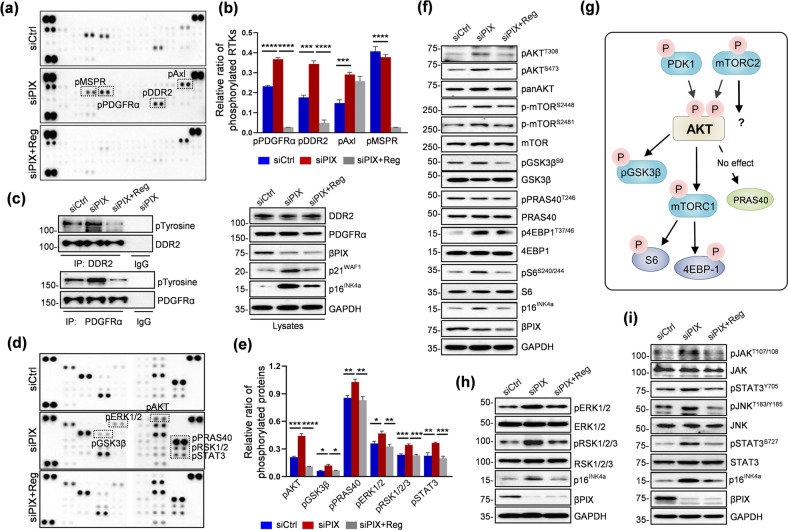
Analysis of regorafenib-responsive receptors and their signaling pathways in βPIX-depleted senescent IMR-90 cells. **a** Representative images of immunoblot membranes for the phospho-RTK antibody array. Responsive receptors are marked (dotted box). **b** Quantification of the optical density of four immunoreactive phosphorylated RTKs. **c** Confirmation of phosphorylated PDGFRα and DDR2 in independent lysates. Left panel, immunoprecipitation of cell lysates with an anti-DDR2 (upper) or anti-PDGFRα (bottom) antibody and immunoblotting with an anti-phospho-tyrosine antibody; right panel, immunoblotting for PDGFRα and DDR2 and of p16^INK4a^ and p21^WAF1^. **d** Representative images of immunoblot membranes for the phospho-kinase antibody array. **e** Quantification of six immunoreactive phosphorylated kinase spots. **f** Confirmation of AKT/mTOR signaling proteins in independent lysates. **g** Schematic diagram of the AKT/mTOR signaling pathway. **h**, **i** Immunoblotting for phosphorylated ERK and RSK (**h**) and phosphorylated JAK/STAT3^Y705^ and JNK/STAT3^S727^ (**i**). For quantification of the Reg-responsive signals in **b** and **e**, the intensities of two spots per protein were measured and normalized to the intensities of the positive control spots. The images are representative of two independent experiments. The error bars indicate the means ± SEMs, *p < 0.05, **p < 0.01, ***p < 0.001, ****p < 0.0001, *t*-test.

To examine the regorafenib-responsive receptor tyrosine kinases and their downstream signaling pathways, a phospho-receptor array analysis was performed. The experiment was performed under conditions similar to those used for the proteomic analysis. Phosphorylation of PDGFRα, discoidin domain receptor (DDR2), and Axl was enhanced by silencing βPIX; among these proteins, PDGFRα and DDR2 but not Axl showed a response to regorafenib (Fig. 4a; quantified in b). A unique response was observed with regard to phosphorylation of the macrophage stimulating protein receptor (MSPR); there was no significant response to siPIX, but in response to regorafenib, its level became undetectable. Immunoblotting was performed using siRNA/regorafenib-treated lysates different from those used in the array to validate this phosphorylation event. Immunoprecipitation of receptors prior to immunoblotting led to intensification of the specific signals. Under the βPIX depletion-induced senescence condition, enhanced phosphorylation of PDGFRα and DDR2 was observed, and this phosphorylation was markedly inhibited by treatment with regorafenib, confirming the array results (Fig. 4c).

Next, we examined the downstream signaling pathway responsible for mediating the receptor phosphorylation-induced events. A phospho-kinase array was used in this experiment. Depletion of βPIX resulted in increased phosphorylation of AKT, glycogen synthase kinase-3β (GSK-3β), proline-rich Akt substrate of 40 kDa (PRAS40), extracellular signal-regulated kinase 1/2 (ERK1/2), ribosomal S6 kinase 1/2/3 (RSK1/2/3), and signal transducer and activator of transcription 3 (STAT3), and all of these phosphorylation events were decreased by treatment with regorafenib (Fig. 4d; quantified in e). Immunoblotting was performed using independent lysates to validate these events. Because increased phosphorylation of AKT, GSK-3β, and PRAS40 suggested the involvement of AKT/mTOR signaling, which plays a central role in senescence (Fig. 4f), this signaling was examined in detail, as shown in Fig. 4g. The increase in βPIX depletion-induced phosphorylation of AKT on T308, a target residue of PDK1, and S473, a target residue of mTORC2, was inhibited by treatment with regorafenib (Fig. 4f). Consistent with this result, a similar response to regorafenib was observed with regard to phosphorylation of GSK-3β on S9; PRAS40; mTOR on S2448 and S2481, the corresponding indicators of mTORC1 and mTORC2 activity; and 4EBP-1 and S6, the downstream effectors of mTORC1. AKT/mTOR signaling under doxorubicin-induced and replicative senescence conditions was further evaluated. Regarding these two types of senescence, treatment with regorafenib resulted in decreased phosphorylation of all of the abovementioned molecules except for 4EBP-1 (Supplementary Fig. 5). Thus, AKT/mTOR signaling was identified as a shared target of regorafenib in the three types of senescence examined. Next, a comparison of regorafenib vs. rapamycin and metformin with this signaling axis as the major target was performed to evaluate the effects of these agents on AKT/mTOR signaling^41,42^. As expected, treatment with regorafenib but not with rapamycin or metformin inhibited activation of AKT (Supplementary Fig. 6). Consistent with this inhibition of AKT, phosphorylation of GSK-3β and PRAS40 was also reduced by treatment with regorafenib. mTORC1 and 2 exhibited differential responses to these drugs; regorafenib exerted effects on both mTORC1 and 2, whereas rapamycin and metformin showed specificity for mTORC1. The extent to which the phosphorylation of the mTORC1 effectors 4EBP-1 and S6 was inhibited was similar after treatment with these agents. Taken together, these results indicate that regorafenib exhibits broad inhibitory activity, including activity toward AKT and mTORC2, compared to rapamycin and metformin.

Because the ERK/RSK axis is located downstream of growth factor receptor/Ras signaling and is thereby activated by phosphorylation, the phosphorylation of related signaling molecules was validated. A similar pattern was observed for the phosphorylation of ERK and RSK, which was increased by depletion of βPIX and decreased by treatment with regorafenib (Fig. 4h), consistent with the array results (Fig. 4e). Activation of JAK/STAT signaling occurs in response to ILs frequently detected as components of the SASP (Fig. 1h). The array results indicated that phosphorylation of STAT3 occurred on S727, which can be phosphorylated by c-Jun N-terminal kinase (JNK), but not on Y705, a target residue of Janus kinase 2 (JAK2). Unexpectedly, the results of immunoblotting indicated that phosphorylation of STAT3 occurred on both S727 and Y705 and that these events showed a response to regorafenib. Consistent with this result, a similar response to depletion of βPIX and treatment with regorafenib was observed for JNK and JAK2, the corresponding upstream kinases (Fig. 4i).

### Regorafenib ameliorated emphysema and attenuated senescence in PPE-treated mice

**Fig. 5:**
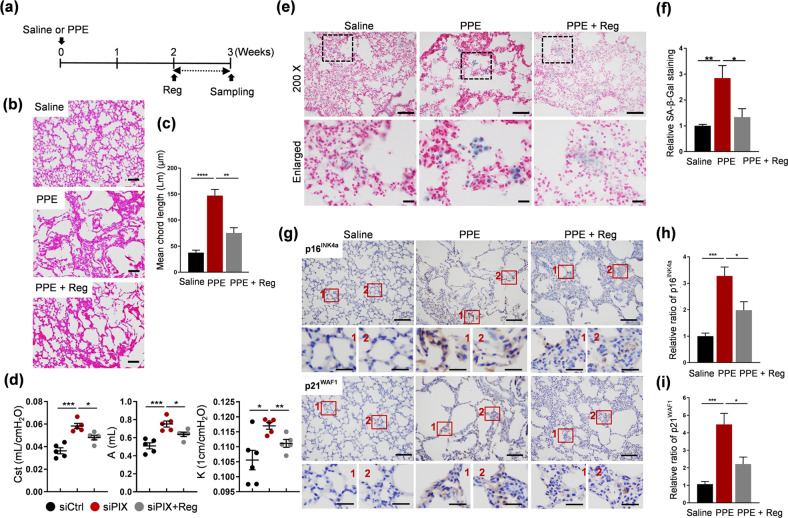
Treatment with regorafenib resulted in amelioration of emphysema and attenuation of senescence in PPE-treated mice. **a** Experimental scheme. **b** Representative H & E images of the lungs. Scale bars, 100 μm **c** Quantification of the mean chord length (Lm) in the three groups. **d** Lung function test. The parameters Cst (static compliance), A (total lung capacity) and K (shape of the deflation limb of the PV loop) are shown. **e** Representative images of SA-β-Gal-stained lungs. **f** Quantification of the data in **e**. **g** Representative images of p16^INK4a^- and p21^WAF1^-stained lungs. **h**, **i** Quantification of p16^INK4a^ (**h**) and p21^WAF1^ (**i**) expression as shown in **g**. Scale bars in **e**, **f**, 100 μm and 20 μm (enlarged). The error bars indicate the means ± SEMs, *N* = 5 mice per group, *p < 0.05, **p < 0.01, ***p < 0.001, *t*-test.

The contribution of senescence to the pathogenesis of chronic lung diseases, including emphysema, is widely accepted^5,43,44^. Therefore, the effect of regorafenib was examined in the model of PPE-induced emphysema in mice. Two weeks after intratracheal instillation of PPE, regorafenib (5 mg/kg) was administered daily for 1 week, as shown in the schematic (Fig. 5a). Treatment with PPE resulted in a significant increase in the mean chord length (Lm), an index of air space enlargement that is compatible with emphysematous changes (Fig. 5b; quantified in c). Treatment with regorafenib resulted in successful suppression of this event, as determined by a marked decrease in the Lm value to ~50% of that of the PPE-treated group. Next, a lung function test was performed using the flexiVent system, which can assess nine parameters, to evaluate the effect of regorafenib on lung function (Fig. 5d, Supplementary Fig. 7). Among the assessed parameters, increases in Cst (static compliance), A (total lung capacity) and K (shape of the deflation limb of the PV loop), which indicate emphysematous changes, were observed in PPE-treated lungs, and significant decreases were then observed in response to treatment with regorafenib. These results indicated a suppressive role of regorafenib in the progression of PPE-induced emphysema in the lungs of mice. We further attempted to determine whether regorafenib is responsible for the regulation of senescence in the model of PPE-induced emphysema (Fig. 5e–i). As reported^44^, treatment with PPE resulted in significant increases in SA-β-Gal activity (Fig. 5e; quantified in f) and the expression levels of p16^INK4a^ and p21^WAF1^ (Fig. 5g; quantified in h and i). Notable declines in SA-β-Gal activity and the levels of p16^INK4a^ and p21^WAF1^ were observed in PPE plus regorafenib-treated lungs. These results indicated a senescence-attenuating role of regorafenib in the model of PPE-induced emphysema in mice.

### Regorafenib reduced senescence-related changes in an animal model of emphysema

**Fig. 6:**
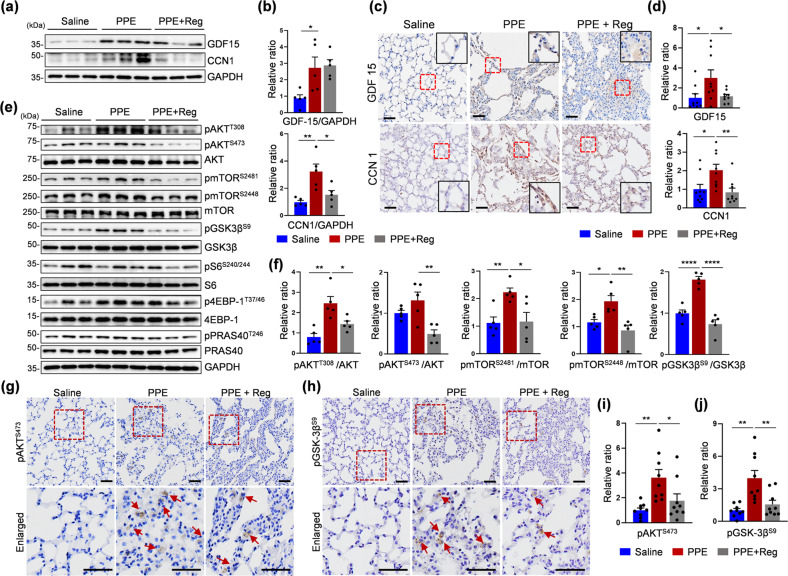
Alterations in the expression of senescence-associated and AKT/mTOR signaling proteins in response to regorafenib in PPE-treated mice. **a** and **e** Representative immunoblots for monitoring the expression of Reg-responsive senescence-associated and AKT/mTOR signaling proteins. **b** and **f** Quantification of the data in **a** and **e**, respectively. *N* = 5 mice per group, *p < 0.05, **p < 0.01, ****p < 0.0001, *t*-test. **c**, **g** and **h** Representative immunohistochemical images of lung tissues. GDF15 (top) and CCN1 (bottom) (**c**), phospho-AKT^S473^ (**g**), and phospho-GSK-3β^S9^ (**h**). **d**, **i** and **j** Quantitative analysis of the immunohistochemical images. GDF 15 (**d**, upper) and CCN1 (**d**, bottom), phospho-AKT^S473^ (**i**), and phospho-GSK-3β^S9^ (**j**). The error bars indicate the means ± SEMs. *N* = 9 mice per group, *p < 0.05, **p < 0.01, *t-*test. Scale bars, 50 μm.

Emphysema is a senescence-related disease; therefore, we attempted to determine whether treatment with regorafenib results in amelioration of senescence-related alterations in the proteome and signaling in a PPE-induced model of emphysema. Upregulation of both GDF15 and CCN1 in PPE-treated mice was found by immunoblotting (Fig. 6a; quantified in b). Treatment with regorafenib resulted in a significant decrease in the expression of CCN1 but not GDF15, whose expression showed wide variation. Therefore, an examination of GDF15 was performed at the cellular level, particularly in alveolar cells, the main cells with cell membrane destruction in emphysema. Increased GDF15 staining was observed along the margin of damaged alveoli (Fig. 6c; quantified in d). The staining intensity of GDF15 in the alveolar region was effectively reduced by treatment with regorafenib (Fig. 6c). The response of CCN1 observed by immunohistochemical staining was similar to that observed by immunoblotting (Fig. 6c; quantified in d).

Next, AKT/mTOR signaling was evaluated as a primary target of regorafenib in PPE-treated mice. Instillation of PPE resulted in increased levels of AKT phosphorylated on T308 and S473, GSK-3β phosphorylated on S9, and mTOR phosphorylated on S2448 and S2481; these levels were decreased by treatment with regorafenib (Fig. 6e; quantified in f). However, in contrast to the results obtained in the IMR-90 model of cell senescence, the phosphorylation of S6, 4EBP-1, and PRAS40 showed no significant response to regorafenib. Immunohistochemistry was performed to evaluate pAKT^S473^ and pGSK-3β^S9^ to understand changes occurring at the cellular level. A small but significant increase in the pAKT^S473^ level was observed after instillation of PPE, and this level was decreased almost to the basal level after regorafenib treatment (Fig. 6g, quantified in i). The level of pGSK-3β^S9^ showed an obvious increase and then a marked decrease in response to treatment with regorafenib (Fig. 6h, quantified in j).

## Discussion

The current study reveals the previously unknown role of regorafenib in cellular senescence. Considering that regorafenib is an anticancer drug, its effect on the attenuation of senescence is highly interesting. In this regard, to suppress the undesired cytotoxic effect of regorafenib, IMR-90 cells and mice were treated with sublethal doses. Therefore, regorafenib can be defined as a senomorphic drug. Senescence is a factor in the pathogenesis of chronic pulmonary diseases, including emphysema. Thus, senotherapy based on senolytics, senomorphics, and their combination has been recognized as a practicable strategy for the treatment of these diseases. One example of a senolytic is dasatinib; a clinical trial of dasatinib in combination with quercetin for the treatment of idiopathic pulmonary fibrosis is currently underway^45^. Treatment with regorafenib resulted in amelioration of senescence and lung function in the model of PPE-induced emphysema. Previous studies have reported on the efficacy of regorafenib in animal models of Alzheimer’s disease and bleomycin-induced fibrosis^30–32^. The role of regorafenib in the attenuation of senescence may provide an explanation for its beneficial effects in these disease models.

Regorafenib exhibited a strong inhibitory effect on diverse types of senescence, suggesting a possible effect on a common signaling pathway. Indeed, treatment with regorafenib resulted in diminished levels of the cell cycle inhibitors p21^WAF1^ and p16^INK4a^, on which the senescence signaling pathway converges^16^, which may provide justification for the restorative effect of regorafenib on proliferation in senescent cells (Fig. 1g). In addition, an in-depth analysis of proteomics data resulted in the identification of two common targets, GDF15 and PAI-1. The findings of this study demonstrated that GDF15 is a secretory factor in senescent cells. Our proteomics data revealed GDF15 as a regorafenib-responsive target. MAPK14 has also been reported as a specific target of regorafenib^46^. In this context, inhibition of the GDF15/MAPK14 pathway with a neutralizing anti-GDF15 antibody resulted in a senomorphic effect in senescent chondrocytes^47^. In addition, GDF15 expression is elevated in many pathological conditions, including COPD and idiopathic pulmonary fibrosis^48^. Therefore, GDF15 is regarded as a potential biomarker of biological aging and ARDs^49^. Secretion of PAI-1 also occurs, and retarded development of senescence and progression of ARDs due to PAI-1 deficiency or inhibitor treatment has been reported^50^. GDF15 and PAI-1 play a causative role in senescence, prompting the development of their inhibitors for clinical use. Their responsiveness to regorafenib may provide a partial explanation for its senescence-attenuating effect. Although CCN1 and IGFBP-3 showed a response to regorafenib under conditions of βPIX depletion-induced senescence, their behavior differed in the other types of senescence examined. CCN1 binds to α6β1 integrin in fibroblasts, which leads to senescence through NADPH oxidase 1-dependent generation of ROS^40^. βPIX knockdown also induces senescence through activation of β1 integrin and ROS generation^13^. They share a similar mechanism for senescence involving β1 integrin activation. Therefore, the elevated CCN1 expression in βPIX knockdown cells and its downregulation by regorafenib can explain the senescence-attenuating effect of regorafenib. It is currently unknown why the elevated CCN1 level did not respond to regorafenib in the contexts of doxorubicin-induced and replicative senescence. Accumulated evidence has demonstrated that IGFBPs, including IGFBP-3, are implicated in cellular senescence^51^. IGFBP-3 expression increases with age and is upregulated in senescent fibroblasts and endothelial cells^52,53^. Our findings in cells with replicative and doxorubicin-induced senescence are in agreement with these reports. Unexpectedly, IGFBP-3 expression was reduced but was restored by regorafenib in the context of βPIX depletion-induced senescence. Long-term treatment with IGF1 also induced senescence accompanied by a reduction in the IGFBP-3 level^54^. Under these conditions, IGFBP-3 may be dispensable for senescence, although deciphering the exact role of the reduced IGFBP-3 expression warrants further study. Together, the distinct responses of CCN1 and IGFBP-3 to regorafenib may reflect the heterogeneity of the types of stressors responsible for inducing senescence. Despite these in vitro findings, the positive response of CCN1 to regorafenib in PPE-treated mice is unique, suggesting the clinical usefulness of regorafenib (Fig. 6a). CCN1, which was identified as a matricellular protein, possesses binding sites for several integrins, including integrin β1; thus, the induction of senescence can occur via this interaction^40^. In addition, a binding site for IGFBP is located at the N-terminus of CCN1, and this interaction might be responsible for modifying the function of IGFBP, resulting in senescence.

Regorafenib, a multityrosine kinase inhibitor, can disrupt the activity of many growth factor receptors. Indeed, treatment of IMR-90 fibroblasts with regorafenib resulted in significant inhibition of activating phosphorylation of PDGFRα and DDR2. The reason that activation of other receptors, such as PDGFRβ and FGFR, cannot occur is unclear. Integrin receptors play pivotal roles in the regulation of cell-matrix adhesion. Aberrant integrin signaling induced by knockdown of βPIX that results in senescence through generation of reactive oxygen species (ROS) has been previously reported^13^. Integrins can interact with many growth factor receptors; therefore, oligomerization and activation of PDGFRα and DDR2 can be achieved^55^. Alternatively, ligand-independent activation of receptors can occur. For example, activation of monomeric PDGFRα can occur via the ROS/Src family kinase pathway without engagement of PDGF^56^; however, there is no indirect mode for activation of PDGFRβ^57^. Phosphorylation and activation of DDR2 by the Src kinase has also been reported^58^. Downstream of these growth factor receptors, the AKT/mTOR and Ras/ERK signaling pathways are two well-known pathways responsible for mediating diverse biological processes, including senescence. Consistent with its effect on the activities of the receptor kinase, treatment of senescent IMR-90 cells with regorafenib resulted in inhibition of activating phosphorylation of AKT and mTOR and a reduction in inhibitory phosphorylation of GSK-3β. In addition, treatment with regorafenib resulted in inhibited phosphorylation of the downstream AKT/mTOR signaling targets PRAS40, 4EBP-1, and S6. Considering the pivotal role of AKT/mTOR signaling in senescence^59^, inhibition of AKT/mTOR signaling can also provide an explanation for the senescence-attenuating effect of regorafenib.

Extensive study of mTORC1 has been conducted in regard to senescence. Although activation of mTORC2 also occurs downstream of phosphoinositide 3-kinase, involving phosphatidylinositol-3,4,5-triphosphate-dependent translocation of mTORC2 to the plasma membrane or mTORC2-ribosome association, the detailed mechanism has not been clearly defined. How does mTORC2 signaling contribute to senescence? (1) Promotion of cellular senescence by TGF-β occurs via multiple effectors, including PAI-I. Activation of mTORC2 can lead to decreased TGF-β signaling through accelerated degradation of Smad2/3^60^. Our proteomics data showed a regorafenib-induced decrease in the levels of TGF-β and PAI-1 (Supplementary Table 2), suggesting that the mTORC2-Smad2/3 axis is a potential target of regorafenib. (2) Silencing of nuclear factor erythroid 2–related factor 2 (Nrf2), a key transcription factor responsible for the execution of an antioxidative stress program, resulted in induction of premature senescence^61^. Suppression of Nrf2 expression by mTORC2 can occur via the AKT-GSK-3β-C/EBPα axis^62^. GSK-3β can also promote cell proliferation in diverse types of cancers. Our finding of inhibition of both cell proliferation (Fig. 1g) and GSK-3β expression (Fig. 4f) in βPIX knockdown cells is in line with the role of GSK-3β. Regorafenib partially reversed these suppressive effects in senescent cells (Fig. 1g, Fig. 4f and Supplementary Fig. 5). Together, these findings indicate that mTORC2 may contribute to senescence attenuation through the AKT-GSK-3β pathway. (3) Activation of NF-*k*B by mTORC2 can also occur and is a critical requirement for SASP induction in an AKT-independent^63^ and AKT-dependent manner via the AKT-IKKα pathway^64^. Overall, the inhibition of mTORC2 as well as mTORC1 by regorafenib might provide an explanation for its prominent effects on the attenuation of senescence.

Findings from a recent study demonstrated mTOR signaling as a causal factor in the senescence of lung cells and emphysema^65^. Our in vitro findings agree with those reported in that study. Therefore, we can speculate that treatment with regorafenib results in amelioration of PPE-induced emphysema, at least in part through attenuation of senescence. The extent of the causal contribution of the effect of regorafenib on senescence to the amelioration of PPE-induced emphysema remains to be determined. However, regarding the phosphorylation of downstream targets of mTORC1, i.e., 4EBP-1, S6, and PRAS40, there is some discrepancy between the in vitro findings and the observations in the PPE model. This might be due to a dominant role of other downstream pathways of mTORC1, such as the IL-1α/NF-*k*B and mitogen-activated protein kinase-activated protein kinase-2 (MK2) pathways, in PPE-treated mice, particularly regarding SASP induction Alternatively, individual heterogeneity may be generated by a complex signaling network in PPE-treated mice, in contrast to the homogeneity of cultured cells.

Findings from accumulated studies have placed mTOR signaling as the central node in both aging and ARDs; thus, the effort to target mTOR signaling has been considerable. Many rapalogs have been developed since the initial discovery of rapamycin as an inhibitor of mTOR^20,66^. mTOR signaling is also a target of metformin and calorie restriction, which result in a prolonged lifespan across a range of model organisms^42,67^. The findings of the current study demonstrated that AKT/mTOR signaling, as a major target, was also inhibited by treatment with regorafenib. Based on the marked senomorphic effect of regorafenib, its combination with other compounds is worthwhile to promote its beneficial effects on emphysema. In addition, there is a temptation to speculate on the therapeutic potential of regorafenib in other senescence-related inflammatory diseases, such as osteoarthritis. This idea is supported by the identification of the SASP components IL-6 and IL-8 and their downstream JAK/STAT3 signaling pathway as targets of regorafenib.

In conclusion, the findings of our study demonstrated that treatment with regorafenib resulted in effective amelioration of both senescence and the progression of emphysema. These findings suggest the potential for the use of regorafenib as a senomorphic drug targeting ARDs.

## Supplementary information

Supplementary information

Supplementary Table 1

Supplementary Table 2
