# Supplementary material for: Defining regorafenib as a senomorphic drug: therapeutic potential in the age-related lung disease emphysema
Source: Exp Mol Med. 2023 Apr 3;55(4):794–805. doi: 10.1038/s12276-023-00966-6 (PMC10167251; doi:10.1038/s12276-023-00966-6)
Supplement: Supplementary file 1 — Supplementary information [file 12276_2023_966_MOESM1_ESM.pdf]

Supplementary Information

Title: Defining regorafenib as a senomorphic drug: therapeutic potential in the age-related lung disease emphysema

Authors: Jung-Jin Park[1,§], Kwangseok Oh[1,§], Gun-Wu Lee[1,§], Geul Bang[2,§], Jin-Hee Park[1], Han-Byeol Kim[1], Jin Young Kim[2], Eun-Young Shin[1,*] and Eung-Gook Kim[1,*]

Affiliation:

[1]Department of Biochemistry, Chungbuk National University College of Medicine, Cheongju, 28644, Korea

[2]Research Center for Bioconvergence Analysis, Korea Basic Science Institute, Ochang, 28119, Korea

Supplementary Materials and Methods

Supplementary Figures and Legends

Supplementary Fig. 1. No cytotoxic and apoptotic activities of regorafenib in IMR-90 cells.

Supplementary Fig. 2. Effect of regorafenib on other types of cellular senescence.

Supplementary Fig. 3. No apoptotic activities of regorafenib in the lungs of mice.

Supplementary Fig. 4. Quality control process for proteome quantification.

Supplementary Fig. 5. Alterations in the AKT/mTOR signaling pathway in response to regorafenib in doxorubicin-induced and replicative senescent cells.

Supplementary Fig. 6. Comparing the effect of regorafenib versus rapamycin and metformin.

Supplementary Fig. 7. Lung function test.

Supplementary Table 1. List of total identified proteins from TMT experiment

Supplementary Table 2. List of total proteins involved in volcano plot analysis of quantified proteins in Reg

Supplementary Materials

(1) Antibody information

Antibody	Use	Dilution	Company/Cat#
pPDGFRα(T754)	WB	1:1000	Cell Signaling Technology/#2992
pPDGFRα(T849)	WB	1:1000	Cell Signaling Technology/#3170
PDGFRα	WB	1:1000	Cell Signaling Technology/#3174
	IP	1:100	
pp90RSK(S380)	WB	1:1000	Cell Signaling Technology/#11989
RSK1/2/3	WB	1:1000	Cell Signaling Technology/#9355
pJAK2 (Y1007,8)	WB	1:1000	Cell Signaling Technology/#3771
JAK2	WB	1:1000	Cell Signaling Technology/#3230
pPRAS40 (T246)	WB	1:1000	Cell Signaling Technology/#13175
PRAS40	WB	1:1000	Cell Signaling Technology/#2691
DDR2	WB	1:1000	Cell Signaling Technology/#12133
	IP	1:100	
pSTAT3(S727)	WB	1:1000	Cell Signaling Technology/#9134
STAT3	WB	1:1000	Cell Signaling Technology/#9139
$p16^{INKa}$	WB	1:1000	BD science/#554079
	IHC	1:200	
$p21^{WAF1}$	WB	1:1000	Santacruze/#sc-6246
	IHC	1:200	
pS6	WB	1:1000	Cell Signaling Technology/#5364
S6	WB	1:1000	Cell Signaling Technology/#2117

p4E-BP1	WB	1:1000	Cell Signaling Technology/#2855
4E-BP1	WB	1:1000	Cell Signaling Technology/#9644
pAKT (T308)	WB	1:1000	Cell Signaling Technology/#13038
pAKT (S473)	WB	1:1000	Cell Signaling Technology/#4060
	IHC	1:150	
pan AKT	WB	1:2000	Santacruze/#4691
GSK-3β	WB	1:1000	Cell signaling Technology/#9832
	IHC	1:200	
pGSK-3β (S9)	WB	1:1000	Cell signaling Technology/#5558
	IHC	1:200	
IGFBP3	WB	1:1000	Santacruze/#sc-365936
GDF15	WB	1:1000	Santacruze/#sc-377195
	IHC	1:50	
CCN1	WB	1:1000	Santacruze/ sc-374129
	IHC	1:200	
Phosphotyrosine	WB	1:1000	Upstate/#05-321
PAI-1	WB	1:1000	Novus/#NBP1-9773
Caspase3	WB	1:1000	Cell Signaling Technology /#9664
GAPDH	WB	1:5000	Invitrogen/#M45-15738
βPIX	WB	1:1000	Shin et al., 2020
	IHC	1:400	Shin et al., 2020
**Secondary Antibody**			
Anti-Mouse IgG HRP	WB	1:5000	Thermo Fisher/#31432
Anti-Rabbit IgG HRP	WB	1:5000	Thermo Fisher/#31212
Anti- Rabbit IgG Biotinylated	IHC	1:200	Vector/BA-1000
Anti- Rabbit IgG Biotinylated	IHC	1:200	Vector/BA-2000

(2) siRNA sequences

siRNA sequences	Company/Reference
Human siPIX	
5’-GGAGGAUUAUCAUACAGAUAGACAA	Thermo Fisher/Shin et al., 2020
Mouse siPIX	
5-*U*GAUGUCAUCCAUGUC*A*C*A	Bioneer/Shine et al., 2020

(*; 2’-O-Me modification)

(3) Primer sequences

Targets	qRT-PCR Primer Sequence (5’ → 3’)
Human IL-8 FW	GGCACAAACTTTCAGAGACAGCAG
Human IL-8 RV	GTTTCTTCCTGGCTCTTGTCCTAG
Human IL-6 FW	AGGAGACTTGCCTGGTGAAA
Human IL-6 RV	GCATTTGTGGTTGGGTCAG
Human IL-1b FW	TGAGCTCGCCAGTGAAATGA
Human IL-1b RV	AGGAGCACTTCATCTGTTTAGGG
Human TIMP-1 FW	CATCCGGTTCGTCTACACCC
Human TIMP-1 RV	GGATAAACAGGGAAACACTGTGC
Human MCP-1 FW	CAGCCAGATGCAATCAATGCC
Human MCP-1 RV	TGGAATCCTGAACCCACTTCT
Human β actin FW	CATGTACGTTGCTATCCAGGC
Human β actin RV	CTCCTTAATGTCACGCACGAT

Supplementary Methods

Screening of an FDA-approved drug library

For screening of senescence-modulating drugs, transfection of IMR-90 cells was performed using siPIX RNAs for 24 h followed by incubation with each drug (0.5 μM) from the library for 24 h [1]. Staining of cells was performed using SA-β-Gal solution, followed by observation under a microscope (Olympus, Japan). Drugs that reduced the staining intensity by less than 50% of siPIX-treated cells were selected in the first round of screening. The second round of screening was conducted, and Western blotting was performed for the cell cycle inhibitors, $p16^{INKa}$ and $p21^{WAF1}$ for confirmation of these candidate drugs.

MTT assay

Transfection of IMR-90 cells was performed using siRNAs for two days. Cells were then treated with regorafenib at the indicated concentrations for 24 h. Cells were incubated in culture medium containing 0.5 mg/ml 3-[4,5-dimethylthiazole-2-yl]-2,5-diphenyltetrazolium bromide (MTT) for 2 h, and in acidified isopropyl alcohol (0.04 N HCl) for extraction of formazan for 30 min. Spectrophotometry was performed at 570 nm for quantification of formazan.

TUNEL assay

Regorafenib (5 or 15 mg/kg) was administrated orally once a day for two weeks in mice (eight weeks old). To perform TUNEL assay, the lungs were prepared to paraffin-embedded sections. TUNEL staining was performed on paraffin-embedded lung sections of mice using TUNEL Assay Kit-BrdU-Red (Abcam) according to the manufacturer's protocol. Paraffin-embedded mouse lung sections were deparaffinized and rehydrated. After washing with PBS, the slides were incubated with TUNEL reaction mixture at 37°C for 1 h, washed with PBS, and incubated with BrdU-Red mixture for 30 min in a humidified chamber. After washing with PBS, the slides were mounted with mounting medium containing DAPI (Invitrogen). Fluorescence images were analyzed with ImageJ software.

TMT-labeling and peptide fractionation

Labeling of trypsin-digested peptides was performed using 10-plex TMT reagent (Thermo Fisher Scientific Inc., MA). Peptide samples (50 µg each) were labeled TMT reagents, respectively. Chemical labeling of peptides with TMT was performed according to the manufacturer's instructions (Thermo Fisher Scientific Inc.). Peptides from siCtrl were labeled with the 126, 127N tag, peptides from siPIX were labeled with the 128C, 129N tag, and peptides from siPIX treated with Reg were labeled with the 129C, 120N tag; 130C and 131 TMT tag reagents were used for labeling the reference samples. For preparation of reference samples, all samples were pooled in the same amount and used for quality control in TMT experiments. Each TMT channel was freshly dissolved in anhydrous acetonitrile (ACN) at a ratio of 0.8:41 (w:v, mg: µL). After incubation at RT for 1 h, the reaction was quenched by addition of 8 µL of 5% hydroxylamine, followed by incubation for 15 min. Then, all TMT-labeled peptides were combined and dried using a Speed-Vac for High-pH fractionation. Fractionation of TMT-labeled peptides was performed by increasing acetonitrile step-gradient elution using a high pH reversed-phase peptide fractionation kit (Thermo Fisher Scientific Inc.). First, acetonitrile and 0.1% trifluoroacetic acid (TFA) were applied for equilibration of the column. Second, the mixed labeled peptide samples and pure water were loaded and then desalted by low-speed centrifugation. Finally, the column was combined with increasing concentrations of high-pH acetonitrile solution. The peptides were subjected to gradient elution of nine fractionations, and vacuum drying was performed on each eluted peptide sample.

LC-MS/MS analysis and quantification

For conduct of proteome analysis, LC-MS/MS analysis was performed using an EASY-nLC 1200 UPLC (Thermo Fisher Scientific Inc.) coupled to a Orbitrap Fusion Lumos Tribrid mass spectrometer (Thermo Fisher Scientific Inc.). Trapping of peptides was performed on a 75 µm × 2 cm C18 precolumn (nanoViper, Acclaim PepMap100, Thermo Fisher Scientific Inc.) prior to separation on an analytical C18 column (75 µm × 50 cm PepMap RSLC, Thermo Fisher Scientific Inc.) at a flow rate

of 250 nL/min. The mobile phases A and B were composed of 0 and 80% acetonitrile containing 0.1% formic acid, respectively. The LC gradient began with 5% B and was maintained at 5% B for 5 min, ramped to 38% B for 90 min, to 95% B for 5 min, and remained at 95% B over 9 min. Finally, it was ramped to 5% B for 1 min. Re-equilibration of the column was performed by application of 5% B for 10 min prior to the next run. Voltage of 2000 V was applied for production of an electrospray. During the chromatographic separation, data-dependent mode was used in operation of the Orbitrap Fusion Lumos, with automatic switching between MS1 and MS2. The following parameters were used in acquisition of MS data: The Orbitrap was used for acquisition of full scan MS1 spectral (400-2000 m/z) with a maximum ion injection time of auto mode at a resolution of 120,000 and an automatic gain control (AGC) target value of 5e4. The Orbitrap mass analyzer was used for acquisition of MS2 spectra at a resolution of 60,000 with high energy collision dissociation (HCD) of 37.5 % normalized collision energy and AGC target of standard mode with a maximum ion injection time of 118 ms. Previously fragmented ions were excluded for 30 sec.

Analysis of MS/MS spectra was performed according to the following software analysis protocol using the Uniprot human database (released on 06-02-2020). The reversed sequences of all proteins were appended into the database for calculation of the false discovery rate (FDR). The peptides were identified using ProLuCID [2] in Integrated Proteomics Pipeline software; IP2 (www.integratedproteomics.com), with a precursor mass tolerance of 10 ppm, and a fragment ion mass tolerance of 100 ppm. Filtering and sorting of the output data files was performed in order to compile a list of proteins with two and more peptide assignments for identification of protein at a false positive rate less than 0.01. TMT reporter ion analysis was performed using Census [3] in Integrated Proteomics Pipeline software within a mass tolerance of 20 ppm. Perseus [4] software (version 1.6.14) was used in performance of statistical analysis. Comparison of protein expression between samples was performed using a multiple sample test with p value set at < 0.05 and hierarchical clustering was constructed for DEPs after calculation of the z-score. A volcano plot was generated after performance of a 2-sample T-test. A list of proteins with log2 fold change of > 0.4 or < -0.4 is shown (Table 1). All

raw MS data files from this study have been deposited in the repository MassIVE with identifier PXD036234.

Supplementary References

1. Shin, E.Y. *et al.* Integrin-mediated adhesions in regulation of cellular senescence. *Sci. Adv.* **6**, eaay3909 (2020).

2. Xu, T., Venable, J. D., Park, S. K., Cociorva, D. ProLuCID, a fast and sensitive tandem mass spectra-based protein identification program. *Mol. Cell Proteomics* **5**, S174 (2006).

3. Park, S. K. *et al*. Census 2: isobaric labeling data analysis. *Bioinformatics* **30**, 2208-2209 (2014).

4. Tyanova, S. *et al*. The perseus computational platform for comprehensive analysis of (Prote)Omics Data. *Nat. Methods* **13**, 731–740 (2016).

Supplementary Figures and legends

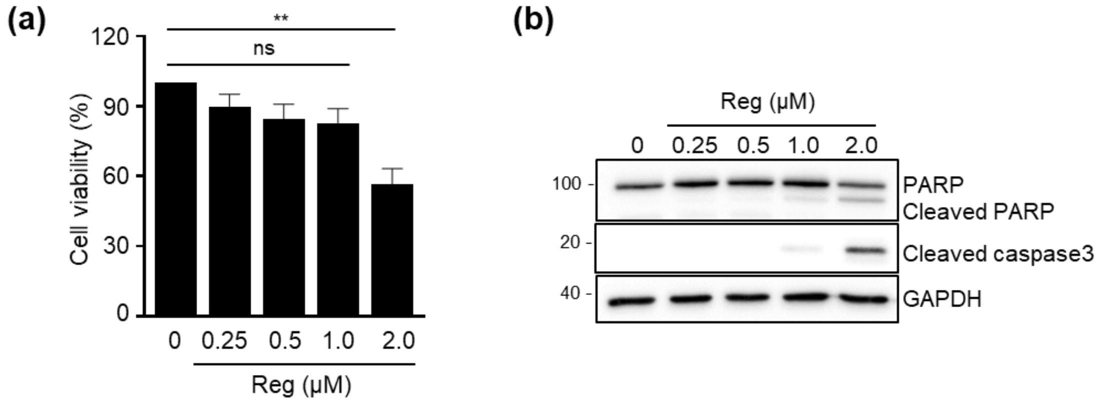

Supplementary Fig. 1. Cytotoxic and apoptotic activities of regorafenib in IMR-90 cells. (a) Quantification of cell viability. Treatment of IMR-90 cells with regorafenib (Reg) was administered at the indicated concentrations for 24 h. Error bars indicate the mean ± SEM from three independent experiments. ns; not significant (concentration of 0 ~ 1 μM, One-way ANOVA), ***P* = 0.0031 (2 μM), *t*-test. (b) Immunoblotting for PARP and caspase 3. Treatment of IMR-90 cells with Reg was administered at the indicated concentrations for 24 h. Immunoblotting of cell lysates was performed using anti-PARP and Caspase 3 antibody, respectively.

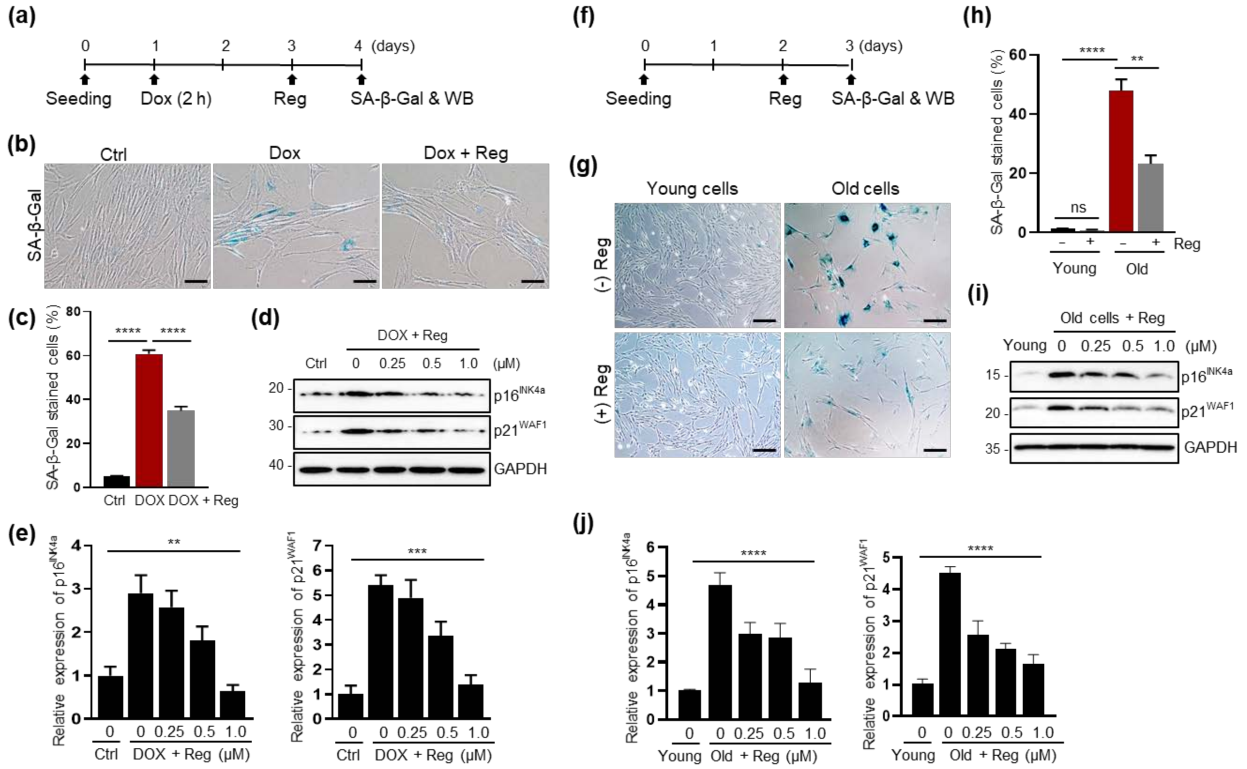

Supplementary Fig. 2. Effect of regorafenib on other types of cellular senescence. (a) Experimental scheme for doxorubicin-induced senescence. IMR-90 cells were treated with 400 nM doxorubicin for 2 h, followed by treatment with Reg (1 μM) or DMSO for 24 h. (b) Images of SA-β-gal staining. Scale bars, 100 μm. (c) Quantification of SA-β-Gal positive cells. n ≥ 200 cells per group from three independent experiments. ****$p < 0.0001$, t-test. (d) Immunoblotting of $p16^{INKa}$, $p21^{WAF1}$, and GAPDH. (e) Quantification of $p21^{WAF1}$ or $p16^{INKa}$ expression levels. **$p < 0.01$, ***$p < 0.001$, One-Way ANOVA. (f) Experimental scheme for replicative senescence (g) SA-β-Gal staining in young (passage #12) and old (passage #43) IMR-90 cells. Scale bars, 100 μm. (h) Quantification of (g). n > 200 cells per group from three independent experiments. **$p < 0.01$, ****$p < 0.0001$, t-test. (i) Immunoblotting for $p16^{INKa}$, $p21^{WAF1}$, and GAPDH in regorafenib-treated old IMR-90 cells. (j) Quantification of (i). ****$p < 0.0001$, One-way ANOVA. Error bars indicate the mean ± SEM.

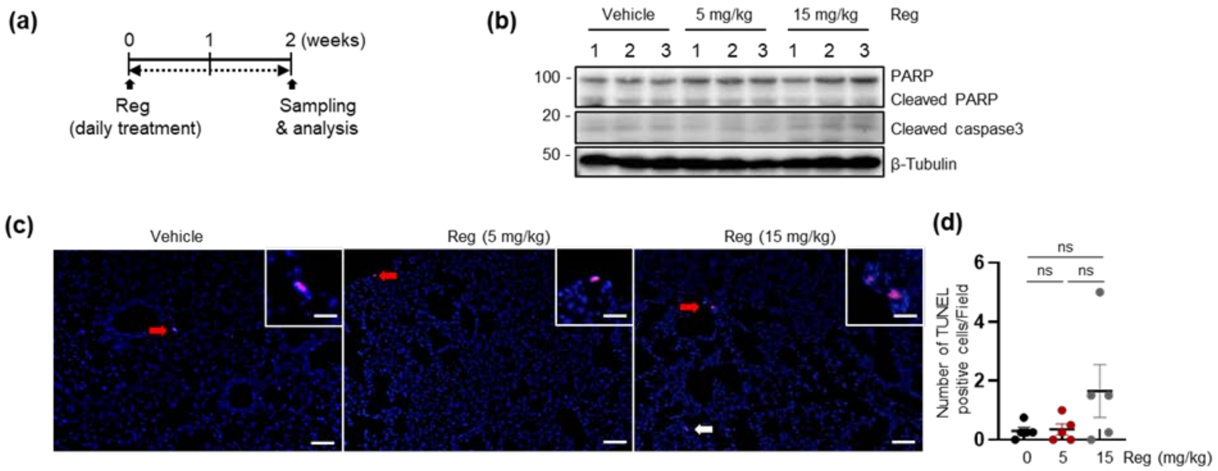

Supplementary Fig. 3. No apoptotic activities of regorafenib in the lungs of the mice. (a) Experimental scheme. Vehicle and regorafenib (5 or 15 mg/kg) were orally administered once a day for 2 weeks. Lungs were collected for Western blotting (b) and TUNEL assay (c). (b) Western blotting with ant-PARP or anti-cleaved caspase 3 antibody in lysates from vehicle- or regorafenib-treated lungs. (c) Representative images of TUNEL assay. (d) Quantification of (c). N = 5 per group. Number of total cells per field < 4,000. Error bars indicate the mean ± SEM, ns = not significant, *t*-test. Scale bars, 100 μm, or 20 μm (boxed).

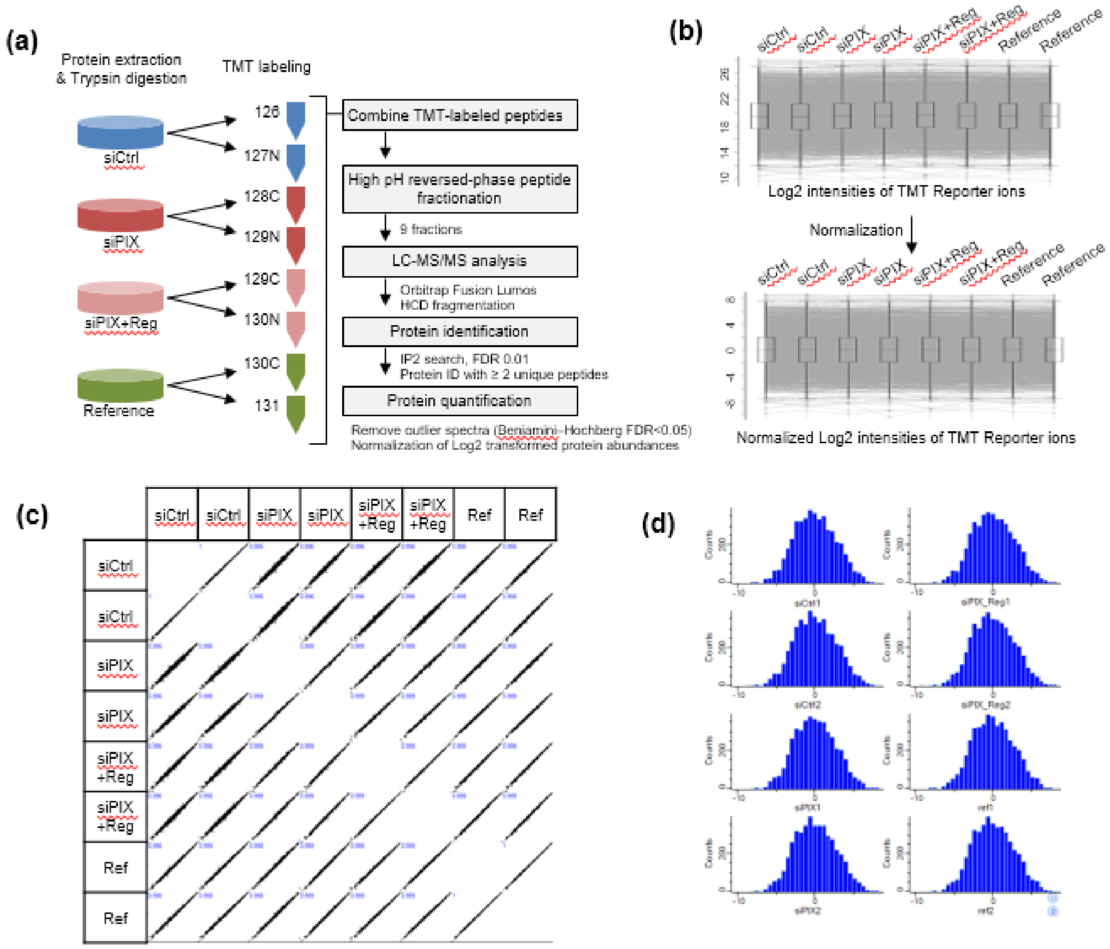

Supplementary Fig. 4. Quality control process for proteome quantification. (a) Overview of TMT-based proteome quantification. (b) Boxplots showing log2 transformed protein abundances calculated according to summarized intensities of TMT reporter ions and normalized log2 transformed protein abundances. (c) Pearson's correlation coefficient between individual samples and references with 4,644 proteins without missing values. (d) Distributions of normalized log2 transformed protein abundances in individual samples and references.

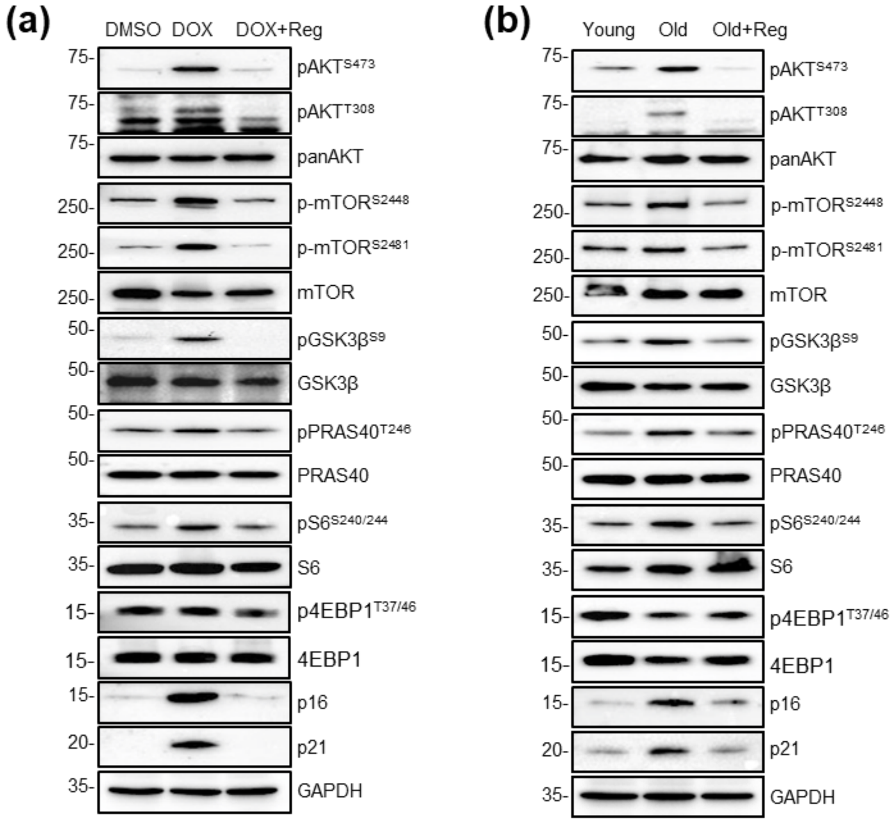

Supplementary Fig. 5. Alterations in the AKT/mTOR signaling pathway in response to regorafenib in doxorubicin-induced and replicative senescent cells. (a) For doxorubicin-induced senescence, IMR-90 cells were treated with doxorubicin for 2 h, followed by washing and incubation with fresh culture media for three days. Cells were incubated with Reg (1 μM) or DMSO for 24 h prior to sampling. Immunoblotting of lysates was performed using the indicated antibodies. (b) For replicative senescence in IMR-90 cells, young and old cells were incubated with or without Reg for 24 h. Immunoblotting of cell lysates for AKT/mTOR signaling proteins was performed. A representative of two independent experiments is shown.

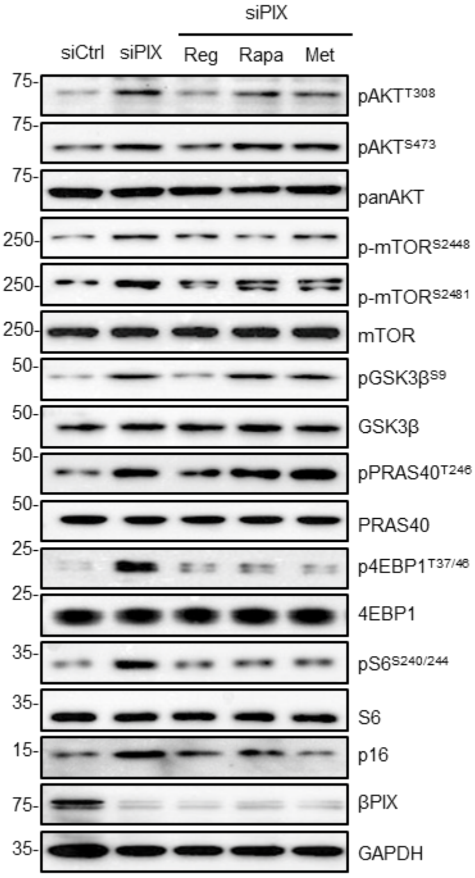

Supplementary Fig. 6. Comparing the effect of regorafenib versus rapamycin and metformin. Transfection of IMR-90 cells was performed using siCtrl or siPIX for 48 h, followed by treatment with Reg (1 μM), rapamycin (Rapa, 20 nM), and metformin (Met, 100 μM) for 24 h. Immunoblotting of cell lysates for AKT/mTOR signaling proteins was performed. A representative of two independent experiments is shown.

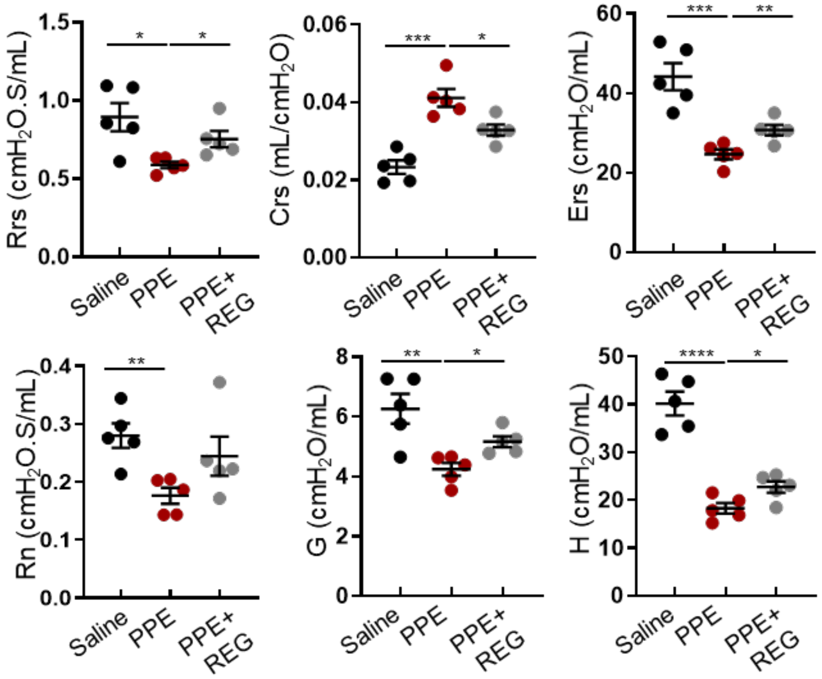

Supplementary Fig. 7. Lung function test. Regarding the perturbation parameters, Rrs (respiratory resistance), Crs (compliance), Ers (elastic resistance), Rn (airway resistance), G (tissue damping), and H (tissue elasticity) are shown. Error bars indicate the mean ± SEM. N = 5 per group, **p* < 0.05, ***p* < 0.01, ****p* < 0.001, *****p* < 0.0001, *t*-test.
